# The effects of a family-centered psychosocial-based nutrition intervention in patients with advanced cancer: the PiCNIC2 pilot randomised controlled trial

**DOI:** 10.1186/s12937-020-00657-2

**Published:** 2021-01-02

**Authors:** Alex Molassiotis, Teresa Brown, Hui Lin Cheng, Angela Byrnes, Raymond Javan Chan, David Wyld, Melissa Eastgate, Patsy Yates, Andrea P Marshall, Rebecca Fichera, Liz Isenring, Ki Fung To, Po Shan Ko, Wang Lam, Yuk Fong Lam, Lai Fan Au, Raymond See-kit Lo

**Affiliations:** 1 School of Nursing, The Hong Kong Polytechnic University, Kowloon, Hong Kong; 2 Royal Brisbane and Women’s Hospital, Brisbane, Australia; 3 School of Nursing and Cancer and Palliative Care Outcomes Centre, Queensland University of Technology, Brisbane, Australia; 4 Division of Cancer Services, Princess Alexandra Hospital, Metro South Health, Brisbane, Australia; 5 School of Medicine, University of Queensland, Brisbane, Australia; 6 School of Nursing and Midwifery, Griffith University, Gold Coast, Australia; 7 Gold Coast University Hospital, Southport, Australia; 8 Nutrition & Dietetics, Bond University, Gold Coast, Australia; 9 Kowloon East Cluster, Hospital Authority, Kowloon, Hong Kong; 10 Haven of Hope Hospital, Tseung Kwan O, Hong Kong; 11 Shatin Hospital, Ma On Shan, New Territories Hong Kong

**Keywords:** Malnutrition, Anorexia, Advanced cancer, Eating-related distress, Quality of life, Caregivers

## Abstract

**Background:**

Malnutrition in advanced cancer patients is common but limited and inconclusive data exists on the effectiveness of nutrition interventions. Feasibility and acceptability of a novel family-based nutritional psychosocial intervention were established recently. The aims of this present study were to assess the feasibility of undertaking a randomised controlled trial of the latter intervention, to pilot test outcome measures and to explore preliminary outcomes.

**Methods:**

Pilot randomised controlled trial recruiting advanced cancer patients and family caregivers in Australia and Hong Kong. Participants were randomised and assigned to one of two groups, either a family-centered nutritional intervention or the control group receiving usual care only. The intervention provided 2–3 h of direct dietitian contact time with patients and family members over a 4–6-week period. During the intervention, issues with nutrition impact symptoms and food or eating-related psychosocial concerns were addressed through nutrition counselling, with a focus on improving nutrition-related communication between the dyads and setting nutritional goals. Feasibility assessment included recruitment, consent rate, retention rate, and acceptability of assessment tools. Validated nutritional and quality of life self-reported measures were used to collect patient and caregiver outcome data, including the 3-day food diary, the Patient-Generated Subjective Global Assessment Short Form, the Functional Assessment Anorexia/Cachexia scale, Eating-related Distress or Enjoyment, and measures of self-efficacy, carers’ distress, anxiety and depression.

**Results:**

Seventy-four patients and 54 family caregivers participated in the study. Recruitment was challenging, and for every patient agreeing to participate, 14–31 patients had to be screened. The consent rate was 44% in patients and 55% in caregivers. Only half the participants completed the trial’s final assessment. The data showed promise for some patient outcomes in the intervention group, particularly with improvements in eating-related distress (*p* = 0.046 in the Australian data; *p* = 0.07 in the Hong Kong data), eating-related enjoyment (*p* = 0.024, Hong Kong data) and quality of life (*p* = 0.045, Australian data). Energy and protein intake also increased in a clinically meaningful way. Caregiver data on eating-related distress, anxiety, depression and caregiving burden, however, showed little or no change.

**Conclusions:**

Despite challenges with participant recruitment, the intervention demonstrates good potential to have positive effects on patients’ nutritional status and eating-related distress. The results of this trial warrant a larger and fully-powered trial to ascertain the effectiveness of this intervention.

**Trial registration:**

The trial was registered with the Australian & New Zealand Clinical Trials Registry, registration number ACTRN12618001352291.

## Background

Inadequate food intake and weight loss, which are associated with risk of malnutrition, frequently occurs among cancer patients. Those at advanced stages of cancer are particularly vulnerable to severe malnutrition due to complex pathophysiological factors including tumor-induced inflammatory responses and metabolic disorders [1]. Studies have reported that 52–61% of advanced cancer patients experience moderate to severe malnutrition [2, 3]. These patients also experience a multitude of nutrition impact symptoms, such as anorexia, fatigue, and dry mouth, which can cause eating-related distress [4]. Similarly, family caregivers can be distressed by the patients’ nutritional problems, which are seldom addressed by healthcare providers [5, 6]. For both patients and caregivers, food is not only important in terms of nutritional value but also in terms of its psychosocial meaning. This psychosocial function is often not well addressed in oncology nutritional care [7].

According to the most recent European Society for Clinical Nutrition and Metabolism guideline, it is recommended that dietary counselling and/or oral nutritional supplements is the first line treatment to prevent and manage malnutrition and includes advice to increase food intake, symptom management and address eating-related distress for advanced cancer patients who are malnourished or at risk of malnutrition [8]. However, such recommendations have not been empirically validated. Current nutrition interventions for advanced cancer patients have focused primarily on provision of dietary advice and/or oral nutritional supplements, and research findings relating to these interventions are inconclusive [9, 10]. Additionally, researchers have attempted to develop and test psychosocial-based nutrition interventions in advanced cancer patients in the past, although effectiveness data have not yet been reported [11, 12].

A qualitative synthesis found that malnutrition in cancer patients has physical, psychological, social and spiritual consequences [13]. Patients reported struggling with weight loss or being pressured by the family to eat [13], they felt they did not receive appropriate dietary advice, and they are often self-managing their weight loss. In addition, cancer patients largely rely on their families for support and family caregivers wish to be actively engaged in the patient’s nutrition care [13]. One study has identified that incorporating family members in the provision of cancer care can have positive effects in reducing anxiety and depression for patients and families [14].

Therefore, a family-centred, psychosocial-based nutrition intervention for advanced cancer patients experiencing or at-risk of malnutrition with the involvement of family caregivers was developed by utilising the team’s expertise in palliative home care for advanced cancer patients and previous qualitative work [13]. The intervention was guided by the Family Systems Theory, with two important features being goal setting and self-regulation [15]. The rationale for family inclusion in nutritional care is that family caregivers can be empowered with nutritional knowledge and skills by healthcare providers in order to optimize their competency and support for the patient and improve adherence to the intervention [15, 16]. Through nutrition education and counselling approaches, patients and family caregivers worked together towards improvement in the patients’ nutritional intake and weight, thereby potentially enhancing quality of life. The intervention, using two different approaches, was successfully tested in a single-arm pre-post experimental study for evaluating its acceptability and feasibility through surveys and interviews with various stakeholders [17, 18]*.*

Building on the success of the feasibility study and informed by the Medical Research Council recommendations for the evaluation of complex interventions [19], the aim of the present study was to assess the feasibility of undertaking a RCT to pilot test outcome measures and to explore preliminary effectiveness before a larger adequately powered randomised controlled trial (RCT) is conducted.

## Methods

### Study design

This study was a two-arm, non-blinded pilot RCT. The experimental group received the family-centred psychosocial-based nutrition intervention, and the control group received usual care involving some nutritional advice and symptom management by the cancer care teams. .

### Sample and settings

The sample included a heterogeneous group of ambulatory patients with advanced cancer, identified at risk of malnutrition, with or without their caregivers at the Royal Brisbane and Women’s Hospital (Australian site) and Haven of Hope Hospital and Shatin Hospital (Hong Kong sites). As there is no standard sample size calculation for a pilot RCT, we planned to recruit 30 subjects (15 per group) to meet the minimal requirement for testing the adequacy of instruments and outcome measurements [20, 21].

### Inclusion criteria

**For patients**, criteria included (1) aged≥18 years old, (2) stage III or IV cancer, (3) with life expectancy of ≥6 months at the opinion of the treating medical oncologist, (4) at risk of malnutrition from any cause (≥2 assessed by the Malnutrition Screening Tool, MST), (5) capable of oral food intake, (6) Eastern Cooperative Oncology Group (ECOG) score 0–2, (7) living at home with a carer (Hong Kong sites) and with or without a carer (Australian site), and (8) able to communicate in English (Australian site) or Chinese (Hong Kong sites) and complete the study questionnaires with or without assistance.

**For caregivers**, criteria included (1) aged≥18 years old, (2) a family member (e.g. husband/wife, children, relatives), or someone who is designated to take care of the patient and who visits for at least 1 h per day on most days, (3) able and willing to provide regular assistance with meals and/or nutritional support at home (ideally being present for 2 or more meals each day), and (4) able to communicate in English (Australian site) or Chinese (Hong Kong sites) and fill in the study questionnaires.

### Exclusion criteria

Patients were excluded if they were (1) completely nil by mouth or participating in other types of nutrition intervention research or receiving enteral/parenteral nutrition; (2) unable to give informed consent and communicate with the study team, (3) currently under the active care of a dietitian with a follow up appointment scheduled.

### Randomisation

The randomisation sequence was generated in advance using a computer-generated randomisation program. The assignment was sequentially numbered in sealed envelopes. Following recruitment and enrolment of participants and obtaining consent for the study, the research assistants at each site liaised with an independent researcher in the team for randomisation allocation. This independent researcher, who was not involved in recruiting patients or delivering the intervention, was responsible for generating the randomisation sequence, accessing the next sealed envelope and then advising the research team to which group the patient had been allocated. The research assistants who were responsible for the data collection were the only persons who remained blinded after assignment to intervention group.

### Procedures

Patients attending the medical oncology cancer care outpatient clinics (Australian site) or outpatient palliative care clinics (Hong Kong sites) were screened for eligibility with the assistance of the treating oncologist and/or clinic nurse. After their medical appointment, the research assistants approached the patients for study briefing and provided them with a detailed information sheet. They discussed the study with the patient/carer to determine if they were interested and completed screening to confirm full eligibility, including completion of the Malnutrition Screening Tool. If the patient/carer met the full inclusion criteria and agreed to join the study, eligible participants were asked to sign an informed consent form prior to randomisation to the intervention or control group. Patients allocated to the intervention group were contacted by the intervention dietitian and were scheduled for the intervention within one to 2 weeks, depending on scheduling practicalities. Patients allocated to the control group received their appointment following usual care referral processes.

### Intervention

Subjects who were randomised to the intervention group received a family-centred nutrition intervention by a dietitian with experience in cancer care and independent of the research team. The intervention provided three structured sessions (2–3 h) of dietitian direct contact time over a 4 week period, inclusive of telehealth (Australian site only) or telephone follow-ups to monitor, reinforce and adjust goals, with a focus on including family/carers in the process. The intervention for this trial was refined based on findings from our prior feasibility intervention study [17, 18]. The context of this intervention was around nutrition impact symptoms, quality of life and food or eating-related psychosocial concerns in patients and caregivers through nutrition counselling, as well as addressing nutrition-related communication between the dyads, rather than solely achieving sufficient energy/protein intake which is a common approach in traditional dietary interventions. The effect of education and nutrition counselling was noted at subsequent reviews and goals modified accordingly. All advice was individualised and goals set in conjunction with the patient +/− the caregiver; this may have included modifications of the diet, altered frequency of meals/snacks and prescription of oral nutrition supplements may have been recommended. Calorie intake was increased through food fortification techniques (with high energy/high protein foods) and/or oral nutrition supplements.

Components of the intervention are shown in Table 1. The intervention also included a culturally-adapted booklet that was provided to the patients and their caregivers (see supplementary file 1 for the English version and supplementary file 2 for the Chinese version of the booklet). If patients were admitted to hospital during the intervention period, the ward dietitian provided dietetic care to the patient whilst they were an inpatient, and the research dietitian continued with the intervention following discharge.

**Table 1:** Intervention content and delivery process

Date	Content	Duration
Week 1, Day 1 (HK); Session 1 (AUS). (Face-to face counselling)	1. Assess patient’s nutrition status, nutrition impact symptoms and diet history including any dietary beliefs	1–1.5 h
	2. Negotiate nutritional goals with patient and caregivers	
	3. Answer any nutrition-related questions from patient and family/carer 4. Provide patient and family-centred nutrition counselling supplemented with relevant nutrition education materials 5. Liaise with multidisciplinary team for management of nutrition impact symptoms as required 6. Make an appointment for telephone/telehealth-based reinforcement counselling	
Week 3, Day 1 (HK); Session 2 (AUS). (Reinforcement counselling via telephone or telehealth)	1. Assess patient’s nutrition status, nutrition impact symptoms and diet history	30–60 min
	2.Identify barriers/facilitators to goal achievement and adjust goal if necessary	
	3. Provide patient and family-centred nutrition counselling	
	4. Make an appointment for telephone/telehealth follow-up	
Week 5, Day 1 (HK); Session 3 (AUS). (Follow-up via telephone or telehealth)	1. Assess patient’s nutrition status, nutrition impact symptoms and diet history	30–60 min
	2. Discuss nutritional goal achievement	
	3.Identify barriers/facilitator to inform future goal setting and adjust goal if necessary	
	4. Provide further patient and family-centred nutrition counselling as required	
	5. Discuss if patient would like ongoing care and refer into usual care as required	

*HK* Hong Kong sites, *AUS* Australian site

### Control group (usual care)

Subjects in the control group received usual care that may have involved some nutrition advice and symptom management. More specifically, in Hong Kong usual care involved nutritional advice and symptom management by the palliative care team in the hospitals. Referral to a dietitian was offered when medically indicated by physicians. An assessment was usually conducted every 4–6 weeks, depending on whether the patient achieved improvements in dietary intake. In Australia, standard care for patients identified with MST>/=2 was referral to a dietitian for nutrition assessment, diagnosis and intervention. This was usually completed within 2 weeks on receipt of referral. Patients were then reviewed as clinically indicated after this initial appointment and/or nursing staff would re-refer if further concerns. In most cases patients usually only received the one appointment (*n* = 8 had 1 appointment, *n* = 5 had 2 and *n* = 2 had 3 appointments). Following completion of outcome assessments, patients were given the option to participate in the intensive dietitian-delivered nutrition counselling off trial.

### Feasibility assessment

Feasibility was assessed using the following measures: recruitment rate (consented rate × 100 divided by screened rate); consent rate (patients); retention rate (patients); proportion of patients with available caregivers; consent rate (caregivers); retention rate (carers), adherence to the protocol; and acceptability of assessment tools (0–10 Likert-type scale measuring how easy and how relevant each questionnaire is; two items).

### Data collection measures

Subjects in both the experimental and control groups were asked to complete self-reported questionnaires at baseline and at their third scheduled session and return via mail with pre-paid envelopes.

#### Socio-demographic and clinical data of patients and family caregivers

Patients’ socio-demographic and clinical data included age, gender, primary cancer site, metastatic sites, co-morbidities, malnutrition screening test score and ECOG score. Caregiver demographics included age, gender, family relationship to patient, employment status, education level, and annual household income.

#### Quality of life (patient)

The 39-item Functional Assessment of Anorexia/Cachexia Therapy (FAACT) scale was used to measure general aspects of quality of life as well as specific anorexia/cachexia-related concerns [22]. The FAACT consists of five subscales including physical wellbeing, social well-being, emotional well-being, functional well-being, and anorexia-cachexia. The Chinese validated version of the FAACT was used in the Hong Kong part of the trial [23]. The Cronbach alpha of this scale in the Hong Kong sample was 0.90 and in the Australian sample 0.86.

#### Nutritional status (patient)

The Patient-Generated Subjective Global Assessment Short Form (PG-SGA-SF) is a validated self-reported nutritional assessment tool assessingweight history, food intake, nutrition impact symptoms, and activities and function [24, 25]. Furthermore, energy/protein intake was measured by using a 3-day food record (Hong Kong sites only, as use of the food record was not viable in the Australian site because of the telephone/telehealth follow-up process it followed). An independent dietitian blinded to subject group allocation estimated energy/protein intake. Weight was measured using a weighing scale provided to each subject on a weekly basis and documented in kilograms (kg; Hong Kong sites only).

#### Eating-related distress (patient)

Two single-item measures were used to assess eating-related distress of patients. One item was about satisfaction with and enjoyment from food, which was selected from the Chinese version of the validated McGill Quality of Life questionnaire [26]. The other item was about distress level related to diet, which is modified based on the item format of the validated Symptom Assessment Scale [27]. These two items were scored on a 0–10 scale, with higher scores indicating more eating-related concerns.

#### Anxiety and depression (caregivers)

The 14-item Hospital Anxiety & Depression Scale (HADS) [28] is a commonly used screening tool to assess anxiety and depression in clinical and community populations. The validated Chinese version of HADS was used in the Hong Kong part of the trial [29]. The Cronbach alpha of this scale in the Hong Kong sample was 0.82 and in the Australian sample 0.85.

#### Self-efficacy (caregivers)

The 21-item Caregiver Self-efficacy Scale (CaSES) assesses self-efficacy for caregivers in people with advanced cancer [30]. The CaSES consists of four domains: Resilience, Self-Maintenance, Emotional Connectivity, and Instrumental Caregiving. The Cronbach alpha of this scale in the Hong Kong sample was 0.92 and in the Australian sample 0.91.

#### Caregiver distress (caregivers)

The Caregiver Distress Checklist is an 18-item caregiver self-assessment scale for evaluating distress when caring for cancer patients. It is a dichotomous scale and each item has either yes or no answer [31]. The Cronbach alpha of this scale in the Hong Kong sample was 0.80 and in the Australian sample 0.395.

#### Eating-related distress (caregivers)

The 19-item Eating-related Distress Scale was used to measure caregiver distress related to advanced cancer patients’ eating problems [5]. The Cronbach alpha of this scale in the Hong Kong sample was 0.71 and in the Australian sample 0.92. In addition, the same single-item about distress level related to eating used by patients was added to the caregiver questionnaire to allow a comparison of patients and caregivers in the level of eating-related distress.

### Data analysis

Data were entered into IBM SPSS Statistics for Windows version 21.0 (Armonk, NY: IBM Corp). Descriptive statistics were used to describe the sample profiles and outcome results (all outcomes were continuous variables except for caregiver distress which was categorical) Non-parametric tests were used to conduct inferential analysis owing to the small sample size. Bivariate analysis was done using Chi-Square or Fisher’s exact tests. Differences in outcomes at the two time points across the two groups were compared using Mann-Whitney U tests. Linear mixed-effects models were used to examine the Group x Time (baseline to final week) interactions on patient and caregiver outcomes. Between-group effect sizes were computed by calculating mean differences of groups with unequal sample size within a pre-post-control design. A *p* value of 0.05 was set as significant level.

### Ethical considerations

Ethics approval was obtained from the Royal Brisbane and Women’s Hospital Human Research Ethics Committee, ref. number HREC/2018/QRBW/43155, and from Kowloon Central/Kowloon East and New Territories East Cluster ethics committees (ref: KC/KE-17-0173-FR-2 and CREC:2017:563). The protocol complied with the Declaration of Helsinki and the ICH-GCP.

## Results

### Sample characteristics

**Fig. 1:**
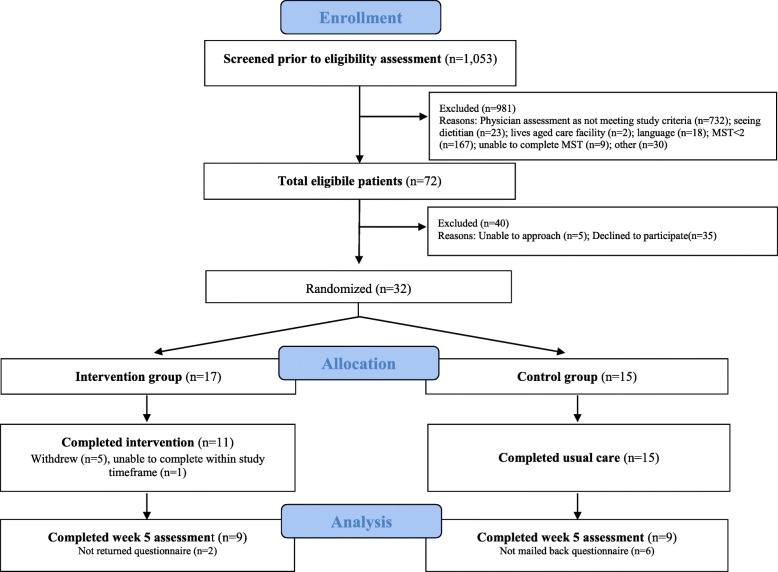
CONSORT recruitment flow diagram (Australian site)

**Fig. 2:**
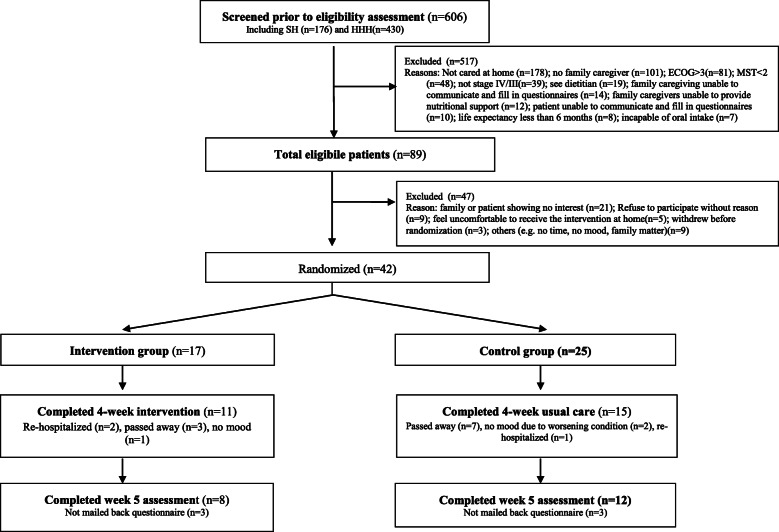
CONSORT recruitment flow diagram (Hong Kong site)

In the Australian site, 32 consenting patients were allocated in the intervention (*n* = 17) and control group (*n* = 15), with 11 and 15 respectively completing the study, and 9 in each group returning by mail the final week assessments (Fig. 1). In Hong Kong, 42 consenting patients were allocated in the intervention (*n* = 17) and control group (*n* = 25), with 11 and 15 respectively completing the study, and 8 and 12 respectively returning by mail the final week assessments (Fig. 2). ECOG performance score was 1 in most patients. Most caregivers were retired (38% in the Hong Kong sample, 46% in the Australian sample) and the majority in both cohorts were of lower income. Sample characteristics were balanced well between the two groups and across sites (Table 2 for patient data and Table 3 for caregiver data).

**Table 2:** Socio-demographic, clinical and baseline characteristics of patients in the Australian and Hong Kong sample (*n* = 74)

Characteristics	Australian sample (*n* = 32)			Hong Kong sample (*n* = 42)		
	All participants (*n* = 32)	Intervention (*n* = 17)	Control (*n* = 15)	All participants (*n* = 42)	Intervention (*n* = 17)	Control (*n* = 25)
Age (years), mean (SD) range	63.7 (14.7) (28–86)	64.6 (15.1) (42–85)	62.7 (14.7) (28–86)	72.4 (14) (35–95)	69.8 (14) (35–89)	74.2 (16) (43–95)
BMI (kg/m^2^), mean (SD) range	26.1 (7.0) (17.6–50.8)	24.4 (3.9) (17.6–31.2)	27.8 (9.0) (18.1–50.8)	19.7 (3.5) (14.5–28.2)	19.9 (3.3) (14.5–28.2)	19.6 (3.6) (15.1–26.4)
Gender	n(%)	n(%)	n(%)	n(%)	n(%)	n(%)
Female	16 (50)	8 (47)	8 (53)	26 (61.9)	11 (64.7)	15 (60)
Male	16 (50)	9 (53)	7 (47)	16 (38.1)	6 (35.3)	10 (40)
Primary cancer type						
Gastrointestinal	8 (25)	2 (12)	6 (40)	20 (47.6)	9 (53.1)	11 (44)
Gynaecological	3 (9)	1 (6)	2 (13)	3 (7.1)	1 (5.9)	2 (8)
Lung	2 (6)	2 (12)	0 (0)	12 (28.6)	4 (23.5)	8 (32)
Skin	4 (13)	3 (18)	1 (7)	–	–	–
Urological	12 (38)	8 (47)	4 (27)	5 (11.9)	3 (17.6)	2 (8)
Other (e.g. breast, thyroid, unknown primary)	3 (9)	1 (6)	2 (13)	2 (4.8)	0	2 (8)
Charlson Comorbidity Index Score, mean (SD) range	6.1 (1.3) (2–8)	5.9 (1.6) (2–8)	6.3 (0.6) (6–8)	–	–	–
Nutritional symptoms						
No appetite	11 (38)	5 (33)	6 (48)	30 (71.4)	10 (58.8)	20 (66.7)
Feeling full quickly	13 (45)	9 (60)	4 (29)	16 (38.1)	5 (29.4)	11 (44)
Dry month	3 (10)	1 (7)	2 (14)	16 (38.1)	6 (35.3)	10 (62.5)
Things taste funny or have no taste	7 (24)	4 (27)	3 (21)	13 (31)	4 (23.5)	9 (36)
Smell bothers me	2 (7)	2 (13)*	0 (0)*	9 (21.4)	3 (17.6)	6 (24)
Nausea	5 (17)	5 (33)	0 (0)	8 (19)	1 (5.9)	7 (28)
Pain	6 (21)	3 (20)	3 (21)	5 (11.9)	2 (11.8)	3 (12)
Constipation	7 (24)	4 (27)	3 (21)	4 (9.5)	3 (17.6)	1 (4)
Diarrhoea	3 (10)	2 (13)	1 (7)			
Problems with swallowing	2 (7)	1 (7)	1 (1)	4 (9.5)	3 (17.6)	1 (4)
Mouth sores	1 (3)	0 (0)	1 (7)			
Vomiting	2 (7)	2 (13)	0 (0)	3 (7.1)	1 (5.9)	2 (8)
PG-SGA-SF total score, mean (SD) range	8.0 (4.0) (1–17)	8 (4) (1–15)	8 (4) (1–17)	10.2 (4.3) (1–19)	9.8 (4.6) (2–19)	10.6 (4.1) (1–19)
Eating-related distress (single item score), mean (SD) range	3.1 (2.9) (0–10)	3 (3) (0–8)	3 (3) (0–10)	3 (3.1) (0–8)	2.6 (3.3) (0–8)	3.2 (3) (0–8)
Eating-related enjoyment (single item score), mean (SD) range	3.8 (3.2) (0–9)	5 (3) (0–9)	3 (3) (0–8)	6.8 (3.2) (0–8)	7.7 (2.9) (0–8)	6.3 (3.4) (0–8)
FAACT total score, mean (SD) range	102.0 (18.8) (60.3–140.0)	96.66 (22.4) (60.3–140.0)	108.2 (11.4) (90.0–127.0)	95.9 (13.6) (65.7–121)	101.6 (15.7)* (74–121)	92.0 (10.5)* (66–114)
FAACT cachexia subscale, mean (SD) range	30.3 (8.0) (20.0–46.0)	29.01 (7.7) (20.0–46.0)	31.69 (8.3) (21.0–45.8)	31 (5.6) (21–42)	32.4 (5.4) (25–42)	30.0 (5.6) (21–40)

*P < 0.05

**Table 3:** Socio-demographic and baseline characteristics of family caregivers in the Australian and Hong Kong sample (*n* = 54)

	Australian sample			Hong Kong sample		
Characteristics	All participants (*n* = 12)	Intervention (*n* = 6)^a^	Control (*n* = 6)^a^	All participants (*n* = 42)	Intervention (*n* = 17)^a^	Control (*n* = 25)^a^
Age (years), mean (SD) Range	58.5 (12.0) (37–71)	57.7 (12.3) (38–71)	59.6 (13.0) (37–70)	57.7 (13.4) (27–84)	69.8 (14) (27–83)	74.2 (14) (29–84)
Relationship to patient	n(%)	n(%)	n(%)	n(%)	n(%)	n(%)
Spouse/partner	8 (67)	4 (67)	4 (67)	21 (50)	12 (70.6)	9 (36)
Children	2 (17)	1 (17)	1 (17)	13 (31)	3 (17.6)	10 (40)
Parents	1 (8)	0 (0)	1 (17)	5 (11.9)	1 (5.9)	4 (16)
Other (i.e.close relatives)	1 (8)	1 (17)	0 (0)	3 (7.1)	1 (5.9)	2 (8)
Gender						
Male	5 (42)	2 (33)	3 (50)	10 (23.8)	5 (29.4)	5 (20)
Female	7 (58)	4 (67)	3 (50)	32 (76.2)	12 (70.6)	20 (80)
Highest education completed						
Elementary school	1 (9)	0 (0)	1 (20)	8 (21.4)	5 (29.4)	3 (16)
Secondary school	4 (36)	3 (50)	1 (20)	24 (57.1)	8 (47.1)	16 (64)
Tertiary education	6 (55)	3 (50)	3 (60)	9 (21.4)	4 (23.5)	5 (20)
Caregiver Distress Checklist, mean (SD) Range	4.6 (3.8) (1–14)	6.2 (4.9) (1–14)	3.0 (1.4) (1–14)	7.3 (3.6) (0–13)	6.5 (3.3) (0–13)	7.76 (3.8) (0–13)
Eating-related Distress (single item) Range	2.7 (3.1) (0–8)	3.8 (3.6) (0–8)	1.3 (1.9) (0–8)	3 (3.1) (0–8)	1.8 (2.6) (0–8)	3.2 (3.3) (0–8)
Eating-related Distress Questionnaire-total score, mean (SD), Range	39.67 (11.3) (23–53)	37.0 (9.8) (23–47)	43.0 (13.5) (23–53)	46.7 (7.3) (28–66)	47.7 (5.7) (38–58)	45.9 (8.7) (28–66)
Hospital Anxiety Depression Scale Anxiety Score, mean (SD) Range	6.9 (4.7) (0–17)	9.4 (4.6) (5–17)	4.4 (3.5) (0–8)	8.9 (3.6) (3–18)	9.40 (4.6) (3–14)	4.40 (3.5) (4–18)
HADS Depression Score, mean (SD) Range	6.3 (4.8) (0–15)	8.4 (4.8) (2–15)	4.2 (4.2) (0–11)	8.9 (4.0) (0–18)	7.9 (4.6) (0–16)	9.5 (3.5) (4–18)
Caregiver Self-Efficacy Scale total score, mean (SD) Range	3.1 (0.5) (2.3–3.95)	3.0 (0.6) (2.3–3.8)	3.27 (0.52) (2.7–3.95)	3.7 (0.5) (2–4)	2.8 (0.5) (2–4)	2.6 (0.5) (2–4)
Instrumental caregiving subscale	3.3 (0.7)	3.0 (0.6)	3.6 (0.7)	2.7 (0.7)	2.8 (0.7)	2.7 (0.7)
Self-maintenance subscale	2.7 (0.9)	2.5 (1.0)	2.9 (0.8)	2.7 (0.5)	2.8 (0.5)	2.7 (0.6)
Emotional-connectivity subscale	3.4 (0.6)	3.1 (0.6)	3.6 (0.4)	2.2 (0.5)	2.4 (0.5)	2.1 (0.5)
Resilience subscale	3.3 (0.5)	3.4 (0.4)	3.1 (0.6)	2.7 (0.6)	2.8 (0.6)	2.6 (0.7)

^a^No statistically significant differences in any of the variables between intervention and control group

### Recruitment feasibility

Over 1000 patients were screened at the Australian site (includes all patients attending general oncology outpatient clinics), to identify 321 potentially eligible patients (i.e. all inclusion criteria met, but MST score still pending), of which 72 patients then met full eligibility criteria (i.e. MST>/=2), and 32 patients consented to participate. For Hong Kong, where the recruitment was in palliative care clinics, just over 600 patients were screened, 89 met full eligibility criteria and 42 patients consented to participate. The patient consent rates were 44% (Australian site) and 47% (Hong Kong sites). The retention rates (patients completing the trial) in the Australian and Hong Kong sites were 81 and 62% respectively, noting that in both sites some of the patients completed the intervention but 14–25% failed to complete the last set of questionnaires and return them to the researchers. The same was the case for the caregivers too; 17% (Australian site) and 14% (Hong Kong site) completed their participation in the trial but failed to return questionnaires. The proportion of patients who had a caregiver in the Australian site was 69%. The Hong Kong sites did not recruit patients who had no caregiver; however, when exploring how many of the patients approached did not have a caregiver, 17% of the screened patients reported so. For more details see Table 4.

**Table 4:** Feasibility aspects of the trial and intervention fidelity

Measure	Australian sample, % (n/N)	Hong Kong sample
**Feasibility**		
Recruitment rate (patients)	10% (32/321)	14.7%
Consent rate (patients)	44% (32/72)	47%
Retention rate (patients completing intervention/trial)	81% (26/32)	62%
Patients returning final assessments	56% (18/32)	48%
Proportion of patients with caregiver	69% (22/32)	100% (inclusion criterion) 83% (from screened sample)
Consent rate for eligible caregivers	55% (12/22)	100% (inclusion criterion)
Retention rate (caregivers completing trial)	84% (10/12)	61%
Caregivers returning final assessments	67% (8/12)	48%
**Intervention fidelity**		
% patients attending all (3) appointments	92% (11/12)	62%
% caregivers attending all (3) appointments	67% (4/6)	62%
Adherence of appointments to timeframe		
• Session 1 (completed within 2–3 weeks from baseline)	42% (5/12)	All appointments were at fixed times through one home visiting and two phone calls for f/u
• Session 2 (completed within 4–6 weeks from baseline)	58% (7/12)	
• Session 3 (completed within 6–8 weeks from baseline)	50% (6/12)	
% patients with treatment goal	100%	100%

### Acceptability of assessment tools

The acceptability of the assessment tools used (0–10-point scale for questionnaire being easy to complete or being relevant) was generally acceptable. In the Australian site patients’ responses for different tools varied from a mean of 6.28–7.52 on a 0–10 scale while for the caregivers’ tools this ranged from 5.18–8.55. In Hong Kong this ranged from a mean of 6.62–6.86 for patients and 6.50–7.07 for caregivers (Table 5).

**Table 5:** Acceptability of the measured questionnaires

	Australian sample		Hong Kong Sample	
Questionnaires	Mean (SD)	Range	Mean (SD)	Range
**Patients**^a^				
PG-SGA-SF: easy	7.52 (2.35)	0–10	6.62 ± 1.64	5–10
PG-SGA-SF: relevant	6.79 (2.69)	0–10	6.76 ± 1.67	5–10
Eating-related distress/enjoyment (single item): easy	7.97 (2.21)	0–10	6.61 ± 1.71	5–10
Eating-related distress/enjoyment (single item): relevant	6.28 (3.18)	0–10	6.86 ± 1.66	5–10
FACCT: easy	7.99 (2.21)	0–10	6.62 ± 1.62	4–10
FACCT: relevant	6.57 (3.04)	0–10	6.81 ± 1.71	5–10
**Caregivers**^a^				
CDC: easy	8.55 (2.16)	3–10	6.86 ± 2.15	0–10
CDC: relevant	6.27 (3.38)	0–10	6.67 ± 1.98	3–10
ERDQ: easy	6.70 (3.62)	1–10	6.79 ± 1.83	4–10
ERDQ: relevant	5.18 (4.24)	0–10	6.79 ± 1.72	4–10
HADS: easy	6.90 (3.45)	1–10	7.07 ± 1.92	3–10
HADS: relevant	6.50 (3.44)	0–10	6.60 ± 2.07	2–10
CaSES: easy	8.09 (2.43)	2–10	6.50 ± 1.98	0–10
CaSES: relevant	6.91 (3.08)	0–10	6.79 ± 2.03	1–10

*PG-SGA-SF* Patient-Generated Subjective Global Assessment Short Form, *FACCT* The functional Assessment of Anorexia/Cachexia Therapy, *CDC* Caregiver Distress Checklist, *ERDQ* Eating-related Distress Questionnaire, *HADS* Hospital Anxiety and Depression Scale, *CaSES* Caregiver Self-Efficacy Scale

^a^29 patients and 11 caregivers completed this assessment in the Australian sample. All patients caregivers completed this assessment in the Hong Kong sample

### Outcome assessments

The Australian data suggest that the patients’ eating-related distress was significantly better over time in the intervention group (*P* = 0.046) than in the control group, showing a large effect size. The same was also the case for the FAACT quality of life scale (*P* = 0.045, large effect size). All other outcome variables had a numerical tendency of improvement in the intervention group that did not reach statistical significance in this small sample, and with small and medium effects sizes (Table 6). For the caregivers’ data, only the Caregiver Distress Checklist outcome improved numerically in the intervention group with a large effect size. Changes in anxiety and depression showed a negligible effect size, while the self-efficacy scale had a small effect size but very small change in scores.

**Table 6:** Nutritional status, eating-related distress/enjoyment, and health-related quality of life of patients at baseline and week 5 in the Australian and Hong Kong sample

Variables	Australian site				Hong Kong site			
	Intervention group (*n* = 9) ^a^ Mean (SD)	Control group (*n* = 9) ^a^ Mean (SD)	Between-group effect size ^b^	*p* value ^c^	Intervention group(*n* = 8) ^a^ Mean (SD)	Control group (*n* = 12) ^a^ Mean (SD)	Between-group effect size ^b^	*p* value ^c^
PG-SGA-SF			0.42	0.38			−0.64	0.16
Baseline	8.89 (1.20)	7.22 (0.80)			9.77 (1.20)	10.56 (0.99)		
Follow up	6.11 (1.50)	6.00 (1.29)			8.70 (1.62)	12.45 (1.28)		
Change from baseline	−2.78 (1.18)	−1.22 (1.28)			−0.94 (1.21)	1.80 (1.42)		
Eating-related distress (single item)			1.02	0.05			−1.00	0.07
Baseline	2.89 (0.75)	2.00 (0.80)			2.61 (0.81)	3.18 (0.65)		
Follow up	2.44 (0.88)	4.00 (0.96)			1.86 (1.13)	5.47 (0.86)		
Change from baseline	−0.44 (0.60)	2.00 (0.96)			−0.81 (1.51)	2.13 (0.76)		
Eating-related enjoyment (single item)			0.41	0.40			−1.07	0.02
Baseline	5.22 (1.15)	2.00 (1.05)			7.55 (0.87)	6.27 (0.70)		
Follow up	7.44 (0.82)	5.89 (0.92)			4.45 (1.15)	6.71 (0.89)		
Change from baseline	2.22 (1.34)	3.89 (1.35)			−3.13 (1.06)	0.46 (0.90)		
FACCT total score			1.09	0.05			0.13	0.93
Baseline	103.54 (7.60)	105.79 (3.96)			101.64 (3.84)	92.05 (3.16)		
Follow up	108.93 (8.20)	99.20 (3.74)			102.21 (5.32)	93.22 (4.20)		
Change from baseline	5.39 (3.12)	−6.58 (4.35)			0.47 (6.27)	1.16 (3.96)		
FACCT cachexia subscale			0.34	0.49			0.72	0.13
Baseline	28.57 (3.04)	32.09 (2.80)			32.09 (1.32)	30.08 (1.18)		
Follow up	32.72 (3.04)	34.05 (2.43)			33.90 (2.34)	32.99 (1.60)		
Change from baseline	4.15 (2.23)	1.96 (2.12)			2.75 (1.15)	2.85 (3.11)		

^a^ Estimated mean and standard error (SE) from linear mixed-effects model

^b^ Effect size for mean differences of groups with unequal sample size within a pre-post-control design

^c^ *P* value for group * time interaction of mean score using linear mixed-effects models

The Hong Kong data showed that patients’ eating-related enjoyment significantly improved in the intervention group (*P* = 0.024, large effect size) while the item of eating-related distress was non-significant (*P* = 0.07) having a near large effect size. All other outcome variables also showed numerically improved values with effect sizes being large (FAACT scale), medium to large (PG-SGA-SF) and near large effect size on the FAACT anorexia-cachexia subscale (Table 6). Weight was maintained in the intervention group and decreased only around 0.5 kg in the control group. However, clinically meaningful improvements were observed in the intervention group in terms of mean energy intake (three-day food diary assessment at week 5 shows 178 kcal more than the same assessment at baseline; large effect size) and mean protein intake (7.5 g change from the three-day food diary assessment at baseline and week 5; near large effect size), the latter reaching *p* = 0.08 at week 3 mid-assessment (Table 7). In the caregivers’ dataset, only the item on eating-related distress had a medium to large effect, while no other outcome variable had any meaningful score change. The caregiver data derives from the Hong Kong sample only, as only 4 caregivers in each completed outcome assessments in Australia, and several had missing data, therefore these were not fully analysed (Table 8).

**Table 7:** Weight and nutritional intake of patients at baseline and week 5 (Hong Kong sample only)

Variables	Intervention group (*n* = 8) ^a^		Control group(*n* = 12) ^a^		Between-group effect size ^b^	*P* value ^c^
Weight (kg)						
Baseline	52.63 (3.72)		52.28 (3.08)			
Week 2	52.22 (3.72)	−0.39 (0.51)	52.27 (3.09)	−0.00 (0.42)		0.418
Week 3	52.04 (3.73)	−0.60 (0.60)	51.92 (3.09)	−0.36 (0.79)		0.193
Week 4	51.54 (3.73)	−1.09 (0.63)	51.58 (3.09)	−0.70 (0.50)		0.267
Week 5	52.80 (3.73)	0.17 (0.64)	51.80 (3.10)	−0.48 (0.50)	0.05	0.148
Energy intake (kcal)*						
Baseline	1078.58 (102.51)		932.14 (88.40)			
Week 3	1168.70 (102.68)	94.24 (115.71)	953.83 (95.35)	23.12 (60.07)		0.115
Week 5	1255.76 (106.45)	182.81 (126.06)	876.69 (99.35)	−43.49 (68.99)	0.77	0.211
Protein intake (g)*						
Baseline	36.93 (3.98)		35.36 (3.46)			
Week 3	40.52 (4.00)	3.63 (3.58)	37.56 (3.69)	2.78 (2.22)		0.084
Week 5	44.46 (4.09)	7.59 (3.72)	34.90 (3.76)	3.24 (2.22)	0.76	0.157

^a^ Estimated mean and standard error (SE) from linear mixed-effects model

^b^ Effect size for mean differences of groups with unequal sample size within a pre-post-control design

^c^ *P* value for group * time interaction of mean score using linear mixed-effects models

*average daily grams or caloric intake from the 3-day food diary

**Table 8:** Caregiver distress, eating-related distress, self-efficacy, anxiety and depression of family caregivers at baseline and week 5

	Intervention group (*n* = 8)		Control group(*n* = 12)		Between-group effect size ^b^	*P* value ^c^
	Estimated mean (SE) ^a^	Change from baseline mean (SE) ^a^	Estimated mean (SE) ^a^	Change from baseline mean (SE) ^a^		
CDC						
Baseline	0.53 (0.12)		0.68 (0.10)			
Week 5	0.56 (0.17)	0.04 (0.23)	0.58 (0.13)	−0.10 (0.18)	−0.29	0.63
Eating-related distress (single item)						
Baseline	1.80 (0.84)		3.18 (0.69)			
Week 5	3.35 (1.14)	1.54 (1.13)	5.27 (0.97)	2.09 (1.24)	−0.74	0.76
ERDQ						
Baseline	47.65 (1.93)		45.92 (1.59)			
Week 5	46.80 (2.68)	−0.68 (2.90)	46.64 (2.12)	−0.28 (1.90)	0.17	0.66
CaSES						
Baseline	2.79 (0.13)		2.62 (0.11)			
Week 5	2.53 (0.16)	−0.28 (0.09)	2.63 (0.14)	0.03 (0.13)	0.08	0.14
HADS_anxiety						
Baseline	8.06 (0.87)		9.44 (0.72)			
Week 5	8.62 (1.08)	0.57 (0.99)	9.69 (0.87)	0.25 (0.81)	−0.49	0.81
HADS_depression						
Baseline	7.98 (0.92)		9.52 (0.73)			
Week 5	10.11 (1.11)	2.12 (1.36)	8.34 (0.89)	−1.16 (0.70)	0.04	0.02

*CDC* Caregiver Distress Checklist, *ERDQ* Eating-related Distress Questionnaire, *CaSES* Caregiver Self-Efficacy Scale, *HADS* Hospital Anxiety and Depression Scale

^a^ Estimated mean and standard error (SE) from linear mixed-effects model

^b^ Effect size calculation was based on the between-group difference in total scores divided by pooled standard deviation

^c^ *P* value for group * time interaction of mean score using linear mixed-effects models

There were no evident associations between patient and caregiver eating-related distress. However, a large number of caregivers (67% in the Australian sample and 62% in the Hong Kong sample) were deemed to experience high distress from caregiving. There were also time by group interaction effects in the patient dataset between body mass index and PG-SGA-SF scale (*p* = 0.018) and eating-related enjoyment (*p* = 0.059, n.s. trend) while such effects were also shown between protein intake and MST score, age and body mass index (*p* < 0.001). There was also a gender effect in the FAACT anorexia/cachexia subscale (*p* = 0.03), with males reporting more anorexia/cachexia. In the caregiver dataset time by group interaction effects were shown between gender (females) and higher eating-related distress (*p* = 0.032) and lower CaSES self-efficacy scale scores (*p* = 0.029). There was also a trend in an effect between eating-related distress and younger age (*p* = 0.059).

## Discussion

This pilot randomised trial follows our previous work in testing the acceptability and feasibility of a family-centred psychosocial-based nutrition intervention and shows that, while it is a difficult trial to recruit patients to, the intervention shows potential in terms of its ability to improve clinical outcomes and quality of life in palliative care patients. The potential benefit, however, was less so in the caregivers. The innovative aspect of this intervention lied not only in the amount of time spent with the patient and the intensiveness of the intervention but also in engaging family caregivers to improve the effectiveness of dietary counselling through maximizing dietary intake, managing nutrition impact symptoms, and reducing eating-related distress, which is different from traditional nutritional interventions that have solely focused on achieving energy and protein requirements.

Recruitment of patients to the trial was a key issue in both countries. Recruitment in general medical oncology clinics showed that 32 patients needed to be screened (primarily through the clinic lists but subsequent eligibility of a smaller number of patients) for every 1 patient recruited, while recruitment in palliative care clinics required 14 patients to be screened for every 1 patient recruited. Issues with recruitment in palliative care settings are common, affected by gatekeeping, ethical considerations, the disease burden experienced by patients, limited life expectancy, the level of complexity of an intervention and patient views on the concept of randomisation [32–34]. Additional factors specific to this trial affecting recruitment were the type of clinic from which patients were recruited, family caregivers being unable to provide nutritional support to their ill relatives, not being cared for at home (requirement for the Hong Kong sites), or cognitive difficulties. Restrictive inclusion criteria, primarily the use of the Malnutrition Screening Tool, was one of the key reasons that recruitment was poor as patients did not always score highly enough on this tool. Perhaps risk of malnutrition (as measured by MST) is not an appropriate way to screen this population of advanced cancer patients, particularly as many of them will be at risk in the future if not now and a more nuanced risk assessment may be needed for the inclusion criteria. It would also be interesting to see if an intervention like the current one is done early for stage 4 patients when patients are well enough to complete it; this could prevent future distress when they are more unwell and less likely to participate in a trial or intervention. Where possible, less restrictive inclusion criteria, addressing patient views on research, simplification of trial complexities and burden in a pragmatic trial design and engaging family caregivers more effectively can improve recruitment and retention rates in a trial.

Only half the patients completed the outcomes assessments for the trial, even though 62–81% completed the actual intervention. While this number is low, it is not dissimilar to other palliative care trials. For example, in a trial of supplement use, nutrition and exercise in advanced cancer patients, 489 patients were screened, 215 were eligible and 53 were recruited, for a recruitment rate of 10.8% [35]; this trial experienced recruitment issues and it did not achieve their expected sample. In another trial of a multimodal nutrition intervention in advanced cancer, the recruitment rate was 11.5%, while compliance with the nutritional supplement component of the trial was 48% [36]. Retention numbers could have been higher in this trial if final assessments were collected in a more timely manner, for example electronically or through a telephone interview, rather than relying on patients/caregivers to return these assessments by mail, which has been also discussed, among other recruitment and retention strategies in international clinical trials, elsewhere [37]. Furthermore, assessment of the dyadic responses to disruption of what Hopkinson [38] describes as ‘food connections’ may be an additional way to identify patient-caregiver dyads that would benefit more from a psychosocial-based nutrition intervention.

Some patients were excluded if they had no caregiver to provide support. As the intervention showed benefits to clinical outcomes of patients, irrespective of carer involvement or not, patients without a caregiver in future trials could also be recruited and offered the intervention. Moreover, while questionnaires were easy to complete, the mean scores indicated some level of difficulty. In particular, the Eating-related Distress scale for caregivers received the lowest score in terms of relevance (=5.18/10) and a future trial should reconsider use of this scale especially as it did not show any change in the caregivers’ scores.

The single items of eating-related distress and eating-related enjoyment showed significant improvements linked with medium to large effect sizes in the patient dataset. The same was the case for the Functional Assessment of Anorexia/Cachexia Therapy. While weight did not change over the 5 weeks of follow-up (a positive outcome nevertheless considering the disease burden), energy and protein intake did improve in a clinically relevant way. For a future trial, eating-related distress or enjoyment and/or the FAACT anorexia/cachexia subscale have demonstrated clinically meaningful outcomes and should be considered as primary outcome candidates. All other scales used were relevant to patients and showed some degree of improvement in the outcomes assessed, with a small to medium effect size. These measures could also be considered as potential trial outcome measures in the future.

To further enhance the impact of the intervention in a future trial, the content of the intervention could further be refined and have stronger elements around some of the areas that these scales depict, such as issues around family/friends pressuring patients to eat, unpleasant taste of foods, or tiredness. Furthermore, the often dynamic meaning attached to nutrition-related problems by patients and their caregivers identified in the literature [39] and the possibility that matched coping styles related to nutrition problems between the dyads can enhance clinical outcomes [40] could further be incorporated into the intervention components.

The results from the caregiver dataset were less convincing. Most outcomes used showed no improvements or change, perhaps with the exception of the Caregiver Distress Checklist, which should be retained in a future trial. However, this scale showed very low reliability coefficients in the Australian sample, and this needs to be carefully evaluated in a future trial. Notwithstanding that the trial was not powered to show differences, there was no specific content for caregivers in the intervention as we expected that improvements in the patients’ outcomes may have also indirectly affected the caregivers. Future work could consider adding components that are more directly relevant to the caregivers. Given the high distress levels reported by caregivers in this study, it is imperative that more focus be directed to managing this distress. The Eating-related Distress scale that the Australian sample identified as the least relevant demonstrated no change over time, however it was an outcome that somewhat improved (not significant) in the Hong Kong caregiver sample and showed a large effect size. This discrepancy may be cultural, as the scale was developed in Japan and may be more relevant to Asian populations than other ethnic groups. If such a focus is included in a future trial, a brief measure may well suffice to decrease the need for a relatively lengthy questionnaire. Eating-related distress is a relatively new concept in palliative care [41] and its role in nutrition care and in improving quality of life should be further considered in future research.

Other limitations of this study include that the participants were a group of advanced cancer patients with different cancer diagnoses and treatment types. As the severity of cachexia may be different across cancer diagnostic groups, future studies should consider focusing on a single cancer diagnostic group. Furthermore, family caregivers were not included in the intervention in one-third of the participants in the Australian site. Hence, the focus in this group of patients was not ‘family-centred’ strictly speaking, although the key principles of the intervention were followed even in the absence of family caregivers. It is also possible, however, that even if the patient did not have a caregiver that wanted to participate in the trial itself, education and counselling may also have been passed onto family members by the patient to help support their nutrition care at home.

## Conclusion

The current pilot trial provided further evidence that a family-centred, psychosocial- based nutrition intervention has the potential to provide benefit to patients at risk of or already experiencing malnutrition. Findings which reflect improvements across multiple outcome measures (quality of life, energy/protein intake, eating-related distress, eating-related enjoyment and nutritional status) provide further support for the intervention. These promising results require confirmation in a fully-powered randomised clinical trial. Considering recruitment and retention issues, a pragmatic trial with more flexible inclusion criteria conducted through multiple sites will be required. Food and eating are culturally bound concepts and as such the content of the intervention also needs to be reflective of cultural realities and preferences.

## Data Availability

Data and materials of the study are available to others upon request.

## Abbreviations

PIcNIC: study acronym; **P**artner**i**ng with families to promote **n**utrition **i**n **c**ancer care
RCT: Randomised controlled trial
ECOG: Eastern Cooperative group
MST: Malnutrition Screening Tool
FAACT: Functional Assessment of Anorexia/Cachexia Therapy
PG-SGA: Patient generated-Subjective global assessment
HADS: Hospital Anxiety & Depression Scale
CaSES: Caregivers Self-efficacy Scale
